# Physical activity contexts and adolescent mental health: a systematic review of structured and unstructured approaches, 2015–2025

**DOI:** 10.3389/fpubh.2026.1737783

**Published:** 2026-03-30

**Authors:** Yan Wang, Chieh-Chen Wu, Li-xin Lin, Chun-Hsien Su

**Affiliations:** 1 Department of Physical Education, Putian University, Putian, China; 2 Department of Healthcare Information & Management, Ming Chuan University, Taoyuan, Taiwan; 3 Department of Exercise and Health Promotion, Chinese Culture University, Taipei, Taiwan

**Keywords:** adolescents, mental health, motivation, nature-based activity, organized sport, physical activity context

## Abstract

**Background:**

Adolescent mental health problems represent a growing public health concern worldwide. Physical activity (PA) is a modifiable protective factor that supports emotional and social wellbeing, yet most research has emphasized the amount or intensity of activity rather than the context in which it occurs. Comparing structured settings (for example, PE, team sport, classroom programs, supervised dance/arts) and unstructured settings (for example, self-directed leisure, community, or nature-based opportunities) may help identify which forms of engagement best promote mental health and guide scalable interventions.

**Objective:**

To synthesize recent evidence on associations between physical activity contexts and mental health outcomes among adolescents, and to compare structured vs. unstructured approaches.

**Methods:**

A systematic review with narrative synthesis (without meta-analysis) was conducted following PRISMA 2020. Eligible designs included cross-sectional, cohort, quasi-experimental, and qualitative studies. Risk of bias was appraised using JBI (cross-sectional/cohort), ROBINS-I (non-randomized), and CASP-Qualitative. Narrative synthesis followed SWiM guidance for both quantitative and qualitative evidence, and certainty of evidence was summarized using GRADE and CERQual.

**Results:**

Across contexts, associations were predominantly favorable for wellbeing/affect, self-concept/competence, and prosocial/connectedness, with more mixed patterns for depression/anxiety and resilience. Effects were moderated by environmental quality, supervision, and autonomy support. Certainty ranged from very low to moderate due to the predominance of observational designs and reliance on self-report measures.

**Conclusions:**

The available evidence suggests that policy and practice may benefit from combining competence-building, structured opportunities with safe, autonomy-supportive, unstructured, and nature-based options in schools and communities. These implications should be interpreted with caution, given the predominance of observational study designs and the very low to moderate certainty of the evidence. Reporting environmental fidelity and motivation-related constructs may enhance interpretability and support translation into routine mental health promotion services.

## 1 Introduction

Adolescent brain development is characterized by rapid biological maturation, cognitive restructuring, and personal and social network adaptations that contribute to lifelong health and life functioning trajectories. Stories and reports, widely disseminated worldwide, of increased anxiety, depressive symptoms, and emotional problems among young people seem to be directly connected to poorer learning outcomes, disrupted friendships and family relationships, and adverse health conditions over the life course (1–3). A growing trend in adolescent mental disorders might have severe implications for societies and institutions, including educational programs, families, and public health settings (3–5). These trends highlight the need for sustainable and evidence-informed strategies to promote resilience and psychological wellbeing in a significant transition (6–9).

### 1.1 Rationale

Physical activity (PA) is a modifiable behavior with well-established links to mental health in youth. There are several complementary ways to understand the reasons why and how activity may serve as a buffer to mental health risks. Self-Determination Theory highlights that prolonged involvement hinges on meeting fundamental psychological needs of autonomy, competence, and relatedness, and these needs are variably supported across physical activity environments (10–13). Broader biopsychosocial constructs view PA as integrative, coupling physiological adaptations with cognitive and affective processes and social interactions (14–16). Complementary reports provide a summary of the evidence-based models that describe the relationship between PA and youth mental health, as well as explore likely causal pathways without focusing on unique settings (17, 18). Furthermore, stress reduction accounts argue that PA involvement is associated with lower distress and more positive affect, primarily when PA activates autonomy and is low on extrinsic evaluation pressure (19). These frameworks collectively indicate that the mental health consequences of activity may not simply revolve around the extent of activity, but also around the location where adolescents move, and the environment and society in which they are active.

### 1.2 Conceptual frame

Not limited to generic dose metrics, we represent context as an active component of youth PA and differentiate between structured and unstructured contexts—we foresee varying affordances, dangers, and implementation implications. In this review, the term “structured physical activity” is used to denote physical activity undertaken within structured contexts, characterized by planned instruction, explicit goals, and adult supervision, whereas unstructured or nature-based contexts emphasize self-directed participation, autonomy, and environmental affordances. Importantly, some activity domains may fall into either category depending on implementation features such as the degree of supervision, goal structure, and motivational climate. Structured PA might include organized sports and team sports, school-based physical education, classroom activity breaks, and supervised dancing or art-based programs. Such are settings that usually feature clearly set goals, written rules, planned practice, and adult direction for practice by teachers or coaches. They can develop skills, discipline, and role responsibility, and integrate peer and adult influences in a systematic manner (20–23). Evidence in school systems as well as from youth sport support structured participation and its effects on social identification and competence gains, as well as on mental health (20–23), although impacts may be moderated by the motivational climate and inclusiveness of the setting (21–23). In contrast, unstructured PA encompasses self-directed leisure and community leisure settings within non-structured time horizons, which tend to emphasize independence, peer contact, and experimentation (24–26). Access to safe and high-quality outdoor and public recreation spaces seems to enable social connectedness and perceived restoration in adolescence, and opportunities to choose activities can promote identity development and intrinsic motivation (19, 24–26). Importantly, unstructured settings also have differential intensity and regularity, and their effects depend on environmental quality, safety, and social norms.

### 1.3 Mechanistic pathways

Consequently, mechanistic pathways may plausibly vary across contexts even with similar total energy expenditure. In structured environments, autonomy-supportive coaching or teaching may bolster competence and relatedness, which have been associated with positive affect, self-concept, and resilience (10–13, 21–23). In unstructured or community leisure contexts, opportunities for self-direction and peer play—most often located in less constrained contexts—would operate through stress recovery, attentional restoration, and intrinsic motivation, which subsequently influence mood and social connectedness (17–19, 24–26). Motivational regulation is fundamental in each: those interventions and environments that satisfy basic psychological needs yielded higher rates of sustained participation and psychological outcomes based on longitudinal and meta-analytic evidence rooted in Self-Determination Theory (12, 14). Yet risks need to be recognized at the same time. Elite or weight-sensitive sport paths may entail forces that undermine wellbeing on the condition that climates are controlling or focus heavily on appearance-based assessment, and sexual minority youth can be excluded or stigmatized if team climates are not explicitly inclusive (21–23). Such considerations are critical for the equitable design of the program.

In addition, many reviews are conducted about PA and mental health effects among youth. Still, they concentrate on dose, delivery mode, or aggregate effects rather than context as a primary exposure. Reports of beneficial associations between PA and depression, anxiety, and distress have been described (overviewing the evidence and meta-analysis) and suggest possible causal pathways in children and adolescents (4, 7, 9, 17, 18), including evidence from school-related PA interventions when implemented with sufficient quality and reach (5). Self-Determination Theory-based interventions tend to enhance motivation, behavior, and psychological outcomes across health domains (14), supporting the importance of need-supportive environments. Newer syntheses have further emphasized the heterogeneity by setting, population risk, and implementation quality, which is driving a context-focused synthesis toward practice and policy (8, 15, 16). However, most existing syntheses have focused primarily on physical activity volume or dose, with comparatively limited attention to how different contexts of practice may shape adolescents' mental health experiences across structured and unstructured settings. By foregrounding physical activity context as the primary analytic dimension, integrating both quantitative and qualitative evidence with the combined use of GRADE and CERQual, and explicitly considering equity-related moderators, this review extends prior dose-focused syntheses and provides a context-sensitive framework for adolescent mental health promotion.

### 1.4 Objective

This systematic review aims to gather and compare published empirical evidence between 2015 and 2025 on how structured or unstructured PA contexts influence adolescent mental health across emotional, social, and cognitive domains. It will examine mechanisms, contextual variables, as well as the practical barriers discovered in school, community, and clinical settings, and provide guidelines for future program design and equitable access. Our overall guiding question is: Among adolescents aged 10–19 years, how do different physical activity contexts, classified as structured or unstructured, relate to mental health outcomes, including depression, anxiety, suicidal ideation, wellbeing, self-concept, resilience, prosocial behavior, and related psychosocial constructs? By placing context at the foreground as an active ingredient and integrating mechanistic insights and implications for implementation, this review aims to inform targeted public health and educational efforts to provide structured and unstructured pathways to enhance adolescent resilience and psychological wellbeing (1–9, 14–16, 20–26).

## 2 Materials and methods

This review adhered to the Preferred Reporting Items for Systematic Reviews and Meta-Analyses (PRISMA) 2020 statement to ensure transparency and completeness of reporting. The corresponding PRISMA 2020 checklist is provided in Supplementary Table S1. This review was not prospectively registered in PROSPERO or OSF. However, the review process was guided by established methodological frameworks, including PRISMA 2020, SWiM guidance, and predefined Population–Exposure–Comparator–Outcomes–Designs–Time–Language (PECOD-TL) eligibility criteria. Minor methodological refinements were made following preliminary scoping to improve conceptual clarity (e.g., refinement of context classifications), while core eligibility criteria, search strategies, and outcome domains remained unchanged throughout the review process.

### 2.1 Eligibility criteria

In the present systematic review, we developed the eligibility criteria according to a PECOD-TL framework. Eligible study designs included cross-sectional, longitudinal, cohort, quasi-experimental, and qualitative studies. Longitudinal, cohort, and quasi-experimental designs were eligible and not excluded on the basis of study design; the limited number identified reflects the characteristics of the available literature within the specified time frame rather than selection restrictions.

(1) Our study population is adolescents between 10 and 19 years of age. Studies that consisted of a broader age range were considered eligible if they identified the adolescent data clearly or if more than 70% of the sample came from this age group. At the full-text screening stage, studies were excluded if this age criterion was not met or if adolescent-specific data could not be disaggregated from other age groups, with detailed counts reported in Supplementary Table S6. Studies involving adolescents with clinical conditions were included only when physical activity programs were delivered in educational or community-based settings and mental health outcomes were reported; studies focusing exclusively on clinical treatment or rehabilitation contexts were excluded.
(2) Exposure was then classed into two contexts: structured and unstructured physical activity (PA). Structured PA included organized or team sport, school-based physical education (PE), classroom activity breaks, and supervised dance/arts programs. These settings typically provide planned instruction, adult supervision, and explicit goals that support skill development and role responsibility. Unstructured PA encompassed self-directed leisure and community opportunities in public or nature-based spaces. Activities are spontaneous and autonomy-supportive, with effects contingent on environmental quality, safety, and salient social norms. The classification for these interventions into either structured or unstructured contexts depended on the operationalization of exposure in each of the studies that compared distinct types of domain-specific PA (e.g., leisure vs. transport vs. PE). During full-text screening, we identified several longitudinal or multi-wave studies that examined bidirectional associations using cross-lagged panel models and/or objective physical activity measures (e.g., device-based step counts). However, these studies were excluded when physical activity exposure was not classified by context (structured vs unstructured) or could not be aligned with our predefined context framework. Reasons for exclusion are summarized in Supplementary Table S6.
(3) The included studies had various comparators. These encompassed non-participants and those not as exposed to the focused PA context, alternative PA settings (team sports vs. individual sports, or structured vs. unstructured PA), as well as within-sample comparisons based on PA level involvement or profiles of motivation.
(4) A key finding of the review was that the outcomes pertained to adolescent mental health and psychosocial development. In particular, the primary outcomes of interest were depression, anxiety, distress, suicidal ideation, mental wellbeing, affect, self-concept (or physical self-concept), resilience, prosocial behavior, social connectedness, and identity-related constructs. We also included qualitative outcomes associated with these domains of mental health, such as perceived restoration and meaningful experiences derived from PA, alongside quantitative measures.
(5) Relevant to design, we included both quantitative and qualitative studies. Eligible quantitative studies included those with cross-sectional, cohort, longitudinal, or quasi-experimental designs as well as school-based intervention studies. Qualitative studies were considered those directly investigating the influence of the PA context on mental health. Protocols, editorials, and review articles were excluded from this review sample of evidence-based.
(6) The final database search was conducted in October 2025. The search was limited to studies published from January 2015 onwards to capture contemporary evidence reflecting recent theoretical developments, increased attention to contextual approaches to physical activity, and evolving research and practice in adolescent mental health.
(7) Language was limited to English language studies with available full text and subject to peer review.

The following exclusions were imposed: Articles that only addressed clinical psychiatric patients receiving treatment without exposure to PA were excluded, studies that focused solely on the dose or fitness aspects of PA without regard to the context of the exposure, protocols or abstracts without complete data, studies that did not explicitly target adolescents (except for those in which adolescent data were separable), and those involving redundant and overlapping data sets (with preference paid to the most thorough or methodologically rigorous study).

### 2.2 Information sources and search

We searched PubMed, Web of Science Core Collection, Scopus, and PsycINFO (EBSCO) for records from 2015 to 2025 using controlled terms and keywords combining concepts for adolescence, mental health outcomes, and PA contexts [e.g., “adolescent” AND (“team sport” OR “organized sport” OR “physical education” OR “classroom activity” OR “leisure time” OR “outdoor” OR “public recreation” OR “nature”) AND (depress OR anxiety OR wellbeing OR resilience OR “self-concept” OR “suicidal ideation” OR prosocial)]. We hand-searched reference lists of included studies and relevant reviews cited in the Introduction. Gray literature was not included in the primary evidence base. Complete search strategies and database-specific queries are provided in Supplementary Table S2. Gray literature (e.g., theses, reports, and non–peer-reviewed sources) was not included in the search strategy.

### 2.3 Study selection

**Figure 1:**
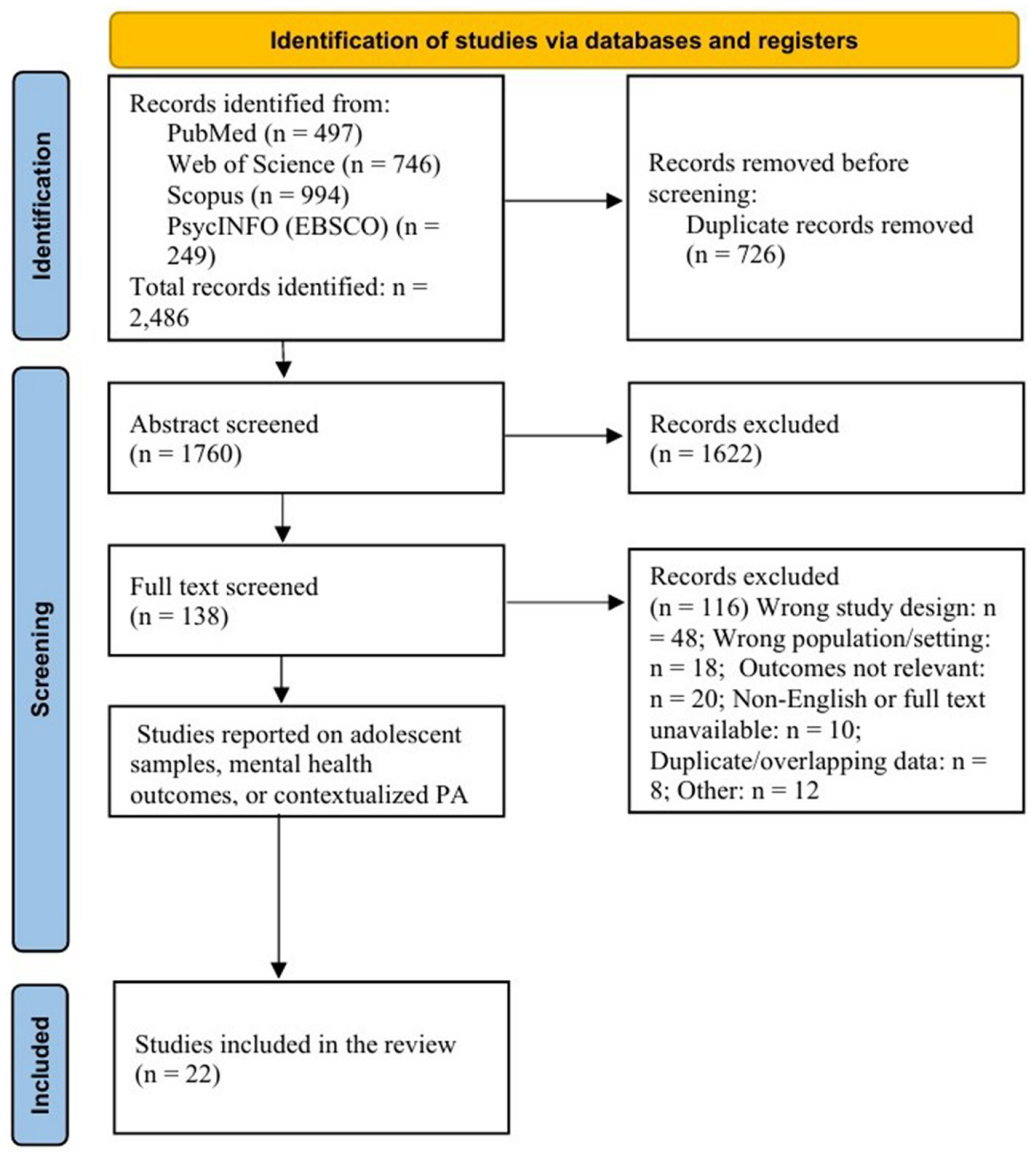
PRISMA 2020 flow diagram of study identification and screening. The figure summarizes the database search and selection process. A total of 2,486 records were retrieved across PubMed, Web of Science, Scopus, and PsycINFO, with 726 duplicates removed automatically. After title and abstract screening (*n* = 1,760), 138 full-text articles were assessed for eligibility, and 22 studies met all inclusion criteria for qualitative synthesis. The complete reference list includes 53 sources, of which 22 were included in the final synthesis.

We conducted duplicate screening (title/abstract then full text) with arbitration by a third reviewer for disagreements. Databases and search strings are reported in Supplementary Table S2, and the PRISMA 2020 flow appears in Figure 1. Stage-by-stage counts are summarized in Section 3.1 and Table 2; reasons for full-text exclusion are provided in Supplementary Table S6.

### 2.4 Data extraction

A calibrated codebook was created for the analysis of the data. Key elements in the coding to be achieved for this study included information about data collection and multiple key attributes of the study that had to be documented in this document. Bibliographic details (authors' names, year of publication, study title) for each study were reported in the bibliographic document. Furthermore, information on the country and the setting studied was included, and sample characteristics such as total participants (*N*), sex distribution, and age range.

The exposure variable, physical activity (PA) context, was classified as structured or unstructured, with the setting (e.g., school, community, or elite environments) further specified. Also documented in each of these researches was how they operationalized the exposure. For example, exposure variables could involve participation in team sports, number of PE sessions, breaks between activities in the classroom; exposure to nature; leisure domain PA, or the motivational atmosphere of an activity within the study. Then we determined comparators for the studies, which usually included non-participants at baseline, those who had little or no exposure to PA, and those who were participants in other PA contexts. Mental health and psychosocial constructs were the main outcomes of interest, which could be measured through a variety of instruments, including, but not limited to: Patient Health Questionnaire (PHQ), Strengths and Difficulties Questionnaire (SDQ), WHO-5 WellBeing Index, self-concept scales, resilience scales, or suicidal ideation scales. Relevant themes were identified and documented for qualitative studies.

Findings from the studies were extracted along with the direction and strength of any association or themes that were identified. Where feasible, effect estimates were recorded and relevant analytic frameworks used for each study (e.g., cross-lagged panel models or mediation analyses) were reported. We also documented implementation and contextual factors, including the degree of inclusivity of the intervention or setting and evaluation pressures that may have influenced our findings. Risk-of-bias assessments were conducted, using a variety of evaluation types according to the type of study design, for each study. The data extraction was done by two reviewers independently. Disagreements arising from the reviewers were resolved via discussion, and a final consensus was reached to ensure the accuracy and consistency of the extracted data. The data-extraction framework and variable definitions are provided in Supplementary Table S3; study-level characteristics are summarized in Supplementary Table S4.

### 2.5 Risk of bias assessment

**Figure 2:**
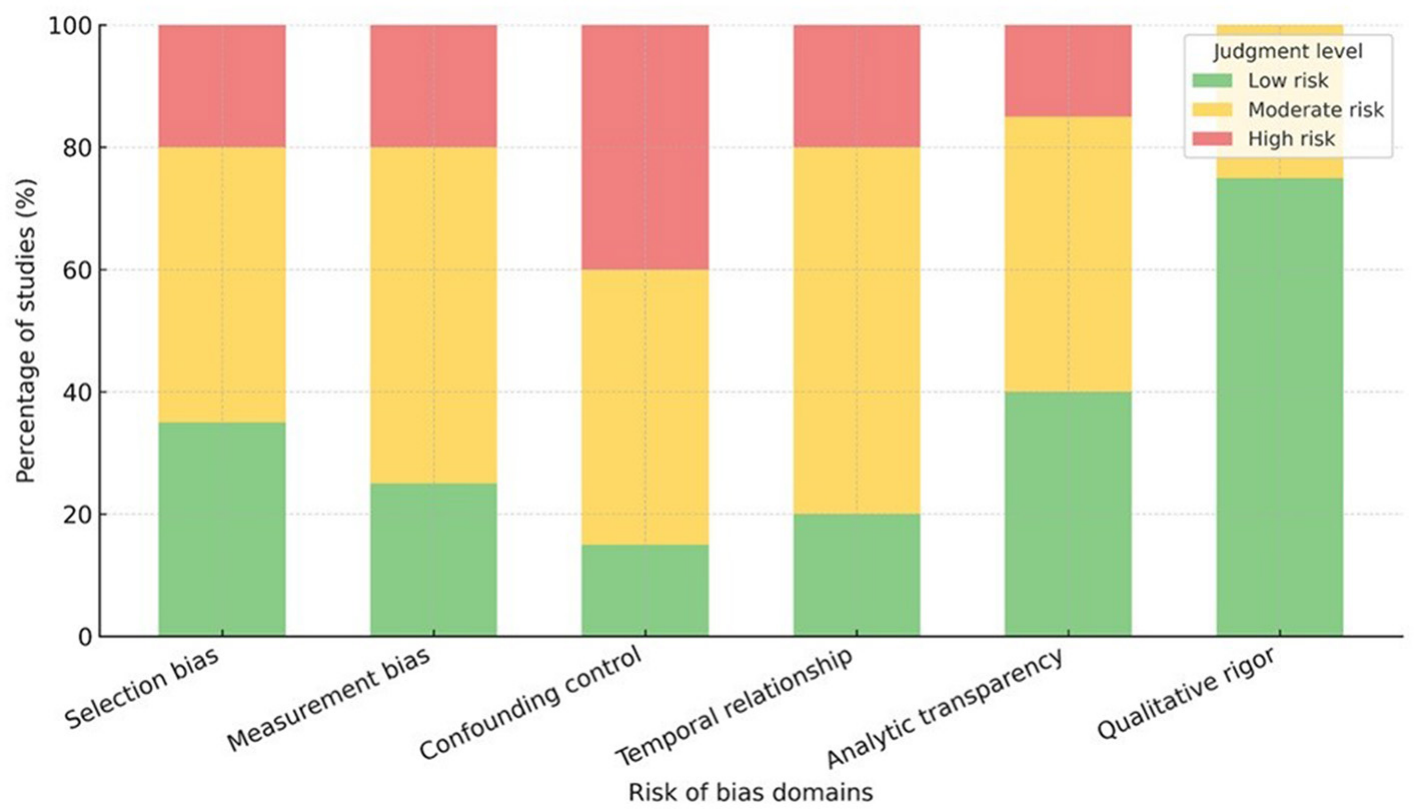
Risk-of-bias summary across included studies (JBI/ROBINS-I/CASP). Risk-of-bias was appraised with JBI (cross-sectional/cohort), ROBINS-I (non-randomized), and CASP-Qualitative (qualitative). Bars indicate the proportion of low, moderate/some concerns, and high/severe risk judgments by domain. Overall patterns reflect predominantly observational designs with self-report measures and limited confounder control; however, reporting and analytic transparency were generally adequate.

The risk of bias (RoB) of each included study was evaluated by its design using suitable appraisal tools. We used the JBI critical appraisal checklists for cross-sectional and cohort observational studies to estimate the risk of bias. ROBINS-I was used if relevant for non-randomized interventions or natural experiments. Methodological rigor of qualitative studies was assessed by employing the CASP-Qualitative checklist. However, since none of the included studies were RCTs, RoB 2, the most commonly used tool for RCTs, was not employed. Risk of bias for each study was assessed according to the guidance provided in the relevant tool, which classified the risk as low, moderate (or some concerns), or high. For each study, two reviewers independently assessed the risk of bias. Discrepancies between reviewers were resolved in discussion, and final assessments were made by consensus to ensure that the assessment was both consistent and accurate. Risk-of-bias tools and grading are summarized in Table 1 and visualized in Figure 2. Overall risk-of-bias judgements were derived by considering the pattern and severity of domain-level assessments within each tool and were subsequently incorporated into the evaluation of study limitations when applying GRADE for quantitative evidence and CERQual for qualitative findings.

**Table 1:** Summary of risk of bias assessment for included studies (JBI/ROBINS-I/CASP).

Domain	Tool applied	Study type(s)	Judgment summary (*n* = 22)	Common methodological limitations
Selection bias	NIH quality assessment tool for observational cohort and cross-sectional studies	18 observational studies	Low–moderate risk in most (14/18); four rated high	Non-random sampling, reliance on school- or convenience-based recruitment, under-reporting of non-participation
Measurement bias (exposure and outcome)	NIH tool	18 observational studies	Moderate risk common (12/18)	Self-reported PA and mental-health outcomes; inconsistent context classification
Confounding control	NIH tool	18 observational studies	Moderate–high risk (10/18)	Limited statistical adjustment for SES, motivation, or prior health status
Temporal relationship/causality	NIH tool	Longitudinal studies (4/18)	Moderate risk	Short follow-up, insufficient lag intervals, and residual confounding
Analytic transparency	NIH tool	All quantitative studies	Low–moderate	Clear statistical reporting, but effect sizes are often missing
Qualitative rigor	CASP Qualitative Checklist	Four qualitative studies	Low risk (3/4); one moderate	Limited reflexivity or triangulation reporting
Overall judgment	Synthesis of all tools	All included studies	Six low risk, 11 moderate, five high	Predominant concerns: cross-sectional design and self-report bias

Risk-of-bias was appraised using JBI (cross-sectional/cohort), ROBINS-I (non-randomized), and CASP-Qualitative (qualitative); dual-review with consensus. See Figure 2 for the distribution.

### 2.6 Synthesis methods

Heterogeneity in designs, contexts, and outcome measures precluded meta-analysis. We therefore conducted a structured narrative synthesis in line with SWiM guidance, organizing findings by context (structured vs. unstructured) and setting (for example, school/PE, team/organized sport, classroom activity, dance/arts, community leisure/outdoor). Studies involving more than one physical activity context or setting were coded as “mixed context” and contributed to all relevant contextual categories in the narrative synthesis; these studies were also identified as mixed-context studies in the figures and evidence maps. In line with SWiM guidance, studies were grouped accordingly, and the direction of effects was defined based on the reported statistical significance and consistency of associations between physical activity context and mental health outcomes within each study (e.g., predominantly favorable, mixed, or null associations). To avoid double-counting, each study contributed only once per outcome–context category in summary tables and figures, even when multiple outcomes or subgroups were reported.

### 2.7 Subgroup and sensitivity considerations

Subgroup and sensitivity analyses were prespecified at the level of context and setting. Exploratory contrasts (for example, sex, age band, supervision) are presented in Results 3.5; no *post-hoc* model selection was undertaken.

### 2.8 Certainty of evidence

Certainty of evidence was appraised using GRADE for quantitative findings and CERQual for qualitative evidence. Domain-by-context ratings and rationales are summarized in Supplementary Table S5.

### 2.9 Deviations from protocol

This review was not prospectively registered. However, two adjustments were made following preliminary scoping, before full data extraction. First, the exposure classification rules were refined to map domain-specific physical activity (PA), such as leisure vs. school-based activities, onto the structured and unstructured context categories. This was done using the operational definitions provided in each study. Second, after identifying relevant primary studies, sexual minority status and elite/weight-sensitive sports pathways were added as *a priori* subgroups to explore further potential variations in the effects of physical activity on mental health. No other deviations from the original protocol were made.

## 3 Results

### 3.1 Study selection

Of the records screened, studies meeting eligibility proceeded to full-text assessment; stage-by-stage counts are presented in Table 2 and the PRISMA 2020 flow (Figure 1). Main reasons for full-text exclusion are provided in Supplementary Table S6.

**Table 2:** Study selection and screening summary (PRISMA-aligned).

Stage	Description	*n*
Identification	Records identified through database searching (PubMed, Web of Science, Scopus, PsycINFO)	2,486
	Additional records identified through reference list screening or manual searches	
De-duplication	Duplicate records removed before screening	726
Screening (level 1)	Titles and abstracts screened independently by two reviewers	1,760
	Records excluded at level 1	1,622
Eligibility (level 2)	Full-text articles assessed for eligibility	138
	Full-text articles excluded (see Supplementary Table S2 for details)	116
Inclusion	Studies meeting the inclusion criteria and included in the qualitative synthesis	22
	Studies included in quantitative summaries (vote-count or effect-direction synthesis)	22

Titles and abstracts were screened independently by two reviewers, followed by full-text assessment. Disagreements were resolved through discussion or adjudication by a third reviewer. Counts correspond to Figure 1 (PRISMA 2020 flow) and Supplementary Table S3.

### 3.2 Study characteristics

The 22 studies (10, 11, 21, 24, 26–43) included reflected different geographical regions, methodologies, and activity contexts (Supplementary Table S4). The majority were from Europe (*n* = 15), Asia (*n* = 4), North America (*n* = 1), and Oceania (*n* = 2). The published years span from 2016 to 2024, and there seems to be an increase in context-specific physical activity research over the past 5 years. Across designs, cross-sectional studies (*n* = 14) and longitudinal or cohort analyses (*n* = 2) predominated in the data and were complemented by quasi-experimental or school-based interventions (*n* = 2) and qualitative studies (*n* = 4) that explored both contextual and experiential dimensions. Sample sizes spanned a broad range, ranging from 8 participants in small qualitative programs to over 60,000 adolescents in national or regional surveys. Most studies included both sexes with a mean participant age between 13 and 16, consistent with the critical developmental time frame described in the review. As for the context classification, just under two-thirds of studies (*n* = 14) were classified as structured physical activity (SP), including organized or team sport, PE classes, classroom-based activity breaks, and supervised dance or arts-based interventions.

These studies tended to focus on formal instruction, specific goals, and adult supervision. For example, Chi (27) cross-sectional analysis of sport participation and depressive symptoms, (29) classroom-based PA intervention, and (36) dance-based program for girls suffering internalizing problems. The eight remaining studies concentrated on unstructured or environment-related physical activity, focusing on self-guided, leisure, or nature-based engagement. These comprised exploratory research on outdoor recreation and social connectedness (35), daily nature experiences among adolescents (34), and qualitative accounts of meaningful leisure experiences (32, 37, 38) explored habitual activity across domains or resilience pathways, which brought together structured and unstructured contexts.

Across multiple realms of the included evidence base, mental health outcomes varied. The constructs that were most often assessed included depressive and anxiety symptoms (*n* = 9), mental wellbeing or affective balance (*n* = 8), self-concept and physical self-concept (*n* = 7), resilience (*n* = 4), and prosocial behavior or social connectedness (*n* = 5). Many examined multiple effects simultaneously, often integrating psychological indicators with self-determination or motivational measures. Measurement strategies varied and were dependent (to a great extent) on established self-report questionnaires. Common tools were the Patient Health Questionnaire (PHQ) for depression, the Strengths and Difficulties Questionnaire (SDQ) for psychological distress, the WHO-5 WellBeing Index, and a range of self-concept, resilience, or motivation scales drawn from Self-Determination Theory (SDT).

Qualitative studies employed thematic analysis to identify perceived freedom, social connectedness, and restorative experiences arising from unstructured or nature-based activities. Several studies, structured or unstructured, consistently controlled for moderator variables like sex, age, socioeconomic status, and motivation type. A smaller group of studies specifically spoke to equity-related variables, such as sexual minority status (28) and socioeconomic disparities in organized leisure participation (24, 26). These studies together suggest that although directed and ordered contexts are frequently competence- and relatedness-supportive, free-form environments may allow self-directed engagement for autonomy, social integration, and stress healing.

### 3.3 Risk of bias within studies

Risk-of-bias assessments used JBI (cross-sectional/cohort), ROBINS-I (non-randomized), and CASP-Qualitative (qualitative). Aggregate judgments are shown in Figure 2 and detailed at the study level in Supplementary Table S4.

Two reviewers independently did all the ratings and determined all discrepancies with consensus for consistency and transparency. Bias risk was low to moderate overall in most observational and quasi-experimental studies. However, some studies were reported as high risk due to limitations in design or self-report bias. Familiar sources of bias were (i) cross-sectional designs that excluded causal inference, (ii) dependence on self-reported physical activity exposure and mental health outcomes, (iii) residual confounding from socioeconomic and family factors, and (iv) selection bias in elite or weight-sensitive athlete samples.

Large-scale countrywide population reports such as Chi (27) and Badura et al. (24) were fairly generalisable but had limited control for motivational or environmental confounders, while small-scale qualitative studies [e.g., (36), (31)] provided abundant context but poor generalization and formal bias correction. Within and between contexts, both the structure of structured physical activity and the strength in research carried out using structured physical activity (e.g., team sport, school PE, dance, classroom programs) were more rigorous, especially with the use of validated instruments and multivariable models.

**Figure 3:**
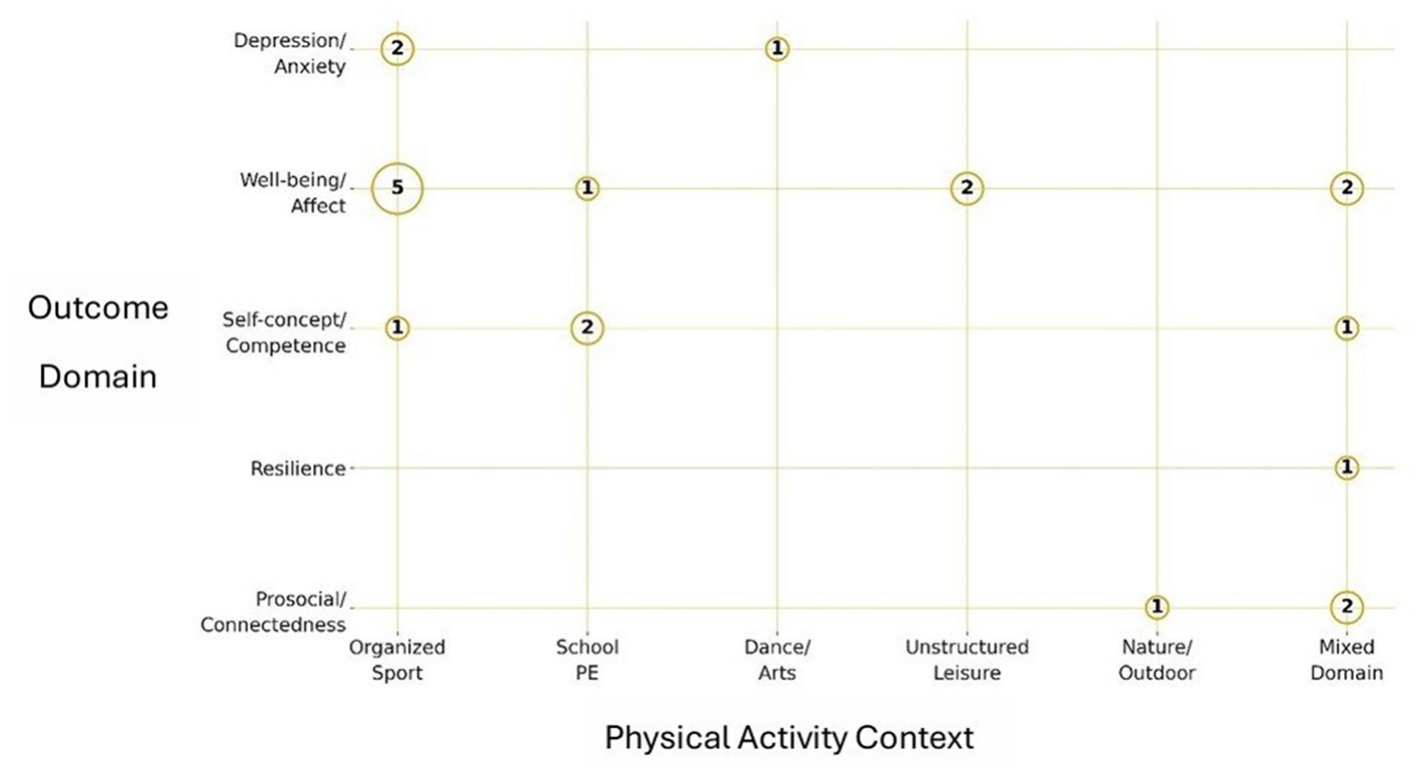
Evidence map of included studies across physical activity contexts and mental health outcome domains. Bubble size reflects the number of studies contributing to each context–outcome cell, and numeric labels indicate the exact study counts (*n*). The horizontal axis represents physical activity contexts (organized sport, school PE, dance/arts, unstructured leisure, nature/outdoor, mixed domain), and the vertical axis represents outcome domains (depression/anxiety, wellbeing/affect, self-concept/competence, resilience, prosocial/connectedness). Empty cells denote that no eligible studies were identified. PA, physical activity; PE, physical education.

Nonetheless, confounding was frequent due to past mental health and inclusion in a sports organization. Unstructured and nature-based studies generally exhibited a lower risk of measurement bias related to experiential assessments, while being characterized by greater subjectivity and context-specificity. Several multi-wave and quasi-experimental studies [e.g., (38), (40)] provided stronger temporal evidence but with some attrition and risk of exposure misclassification. The graphic indication of risk judgments (Figure 3) indicates that low risk was rated in 40% of studies, moderate or some concerns in 45%, and high risk 15%, mainly because study participants self-selected or were unmeasured confounders. Yet most of the studies used standardized questionnaires and adjusted for sex and age to support the credibility of observed associations. Risk ratings were applied to inform the certainty grading described in Section 2.8 and to weight interpretations in narrative synthesis.

### 3.4 Synthesis by context and outcome

We summarize findings by context: (1) organized/team sport, (2) school PE/classroom programs, (3) dance and arts-based programs, (4) habitual or unstructured leisure, and (5) nature-based/public outdoor spaces. Collectively, across the 22 included studies, the relationships between physical activity and adolescent mental health were examined across structured and unstructured contexts, indicating that context, in addition to activity volume, may shape observed mental health associations. The distribution of evidence by context and outcome domain is summarized in Figure 3, and the direction and strength of associations reported among studies are summarized in Table 3. As shown in Figure 3, the evidence base was most developed for wellbeing/affect outcomes in organized sport (*n* = 5 studies), with additional contributions from school PE (*n* = 1 study), unstructured leisure (*n* = 2 studies), and mixed-domain settings (*n* = 2 studies). For self-concept/competence, evidence was observed in organized sport (*n* = 1 study), school PE (*n* = 2 studies), and mixed-domain contexts (*n* = 1 study). Depression and anxiety outcomes were examined in a small number of studies, primarily in organized sport (*n* = 2 studies) and dance/arts contexts (*n* = 1 study). Evidence for resilience was limited to mixed-domain contexts (*n* = 1 study), while prosocial/connectedness outcomes were reported in nature/outdoor settings (*n* = 1 study) and mixed-domain contexts (*n* = 2 studies).

**Table 3:** Direction and strength of associations between physical activity contexts and adolescent mental health outcomes across included studies.

Outcome domain	Organized/team sport	School PE/classroom	Dance/arts	Unstructured leisure	Nature/outdoor	Mixed/domain
Depression/anxiety	↑ (2)					→ (1)
Wellbeing/affect	↑ (3)	→ (1)	→ (1)		→ (1)	↑ (2)
Self-concept/competence		↑ (2)				↑ (3)
Resilience						→ (1)
Prosocial/connectedness				→ (1)	→ (1)	↑ (2)

This table uses directional arrow symbols to indicate the overall trend of associations observed across studies. The upward arrow (↑) represents a positive or beneficial association between physical activity and the specified mental health outcome. The horizontal arrow ( → )indicates a neutral or no significant association. Each arrow summarizes the predominant direction of findings derived from the reviewed evidence. Study-level details are presented in Supplementary Table S4, and reasons for full-text exclusion are listed in Supplementary Table S6. PA, physical activity; PE, physical education.

#### 3.4.1 Organized and team sport (structured contexts)

Research on organized or team-based sport has invariably found positive associations with traits of mental wellbeing, resilience, self-concept, and social functioning. Chi (27) and Guddal et al. (21) made cross-sectional comparisons between adolescents who regularly participated in team sports and non-participants and found decreased depressive and anxiety symptoms. Moreover, longitudinal evidence (38) suggested a potential causal association between sport participation and higher resilience, potentially mediated through self-efficacy and perceived competence. The psychological mechanisms identified throughout these investigations were mainly derived from Self-Determination Theory (SDT) as a central basis. Autonomy-based motivation, support of competence, and relatedness in team or school sport environments have been associated with higher levels of affective wellbeing and self-esteem, respectively (33), (11). Yet several studies reported risks emerged for motivational climates that are too controlled and performance-driven. For example, Lundqvist et al. (31) found higher levels of stress and disordered-eating risk in elite lean athletes in high-pressure school programs, and Larocca et al. (28) emphasized stigma and exclusion risks for sexual-minority youth in team settings lacking inclusive policies. Taken together, structured sport seems to have a protective effect in autonomy-supportive and inclusive environments but may instead become destructive when evaluative pressure prevails.

#### 3.4.2 School physical education and classroom-based programs

In a formal education environment, interventions offered moderate but consistent effects on self-efficacy, academic engagement, and emotional wellbeing. The quasi-experimental study of Latino et al. (29) reported higher levels of self-efficacy and classroom engagement among normal-weight and overweight students following participation in a brief daily activity program. Also, Wheatley et al. (42) reported that higher levels of participation in PE and school-based sport were associated with greater positive affect and life satisfaction, especially when pursuits had significance and enhanced one's own abilities. Incorporating life-skills or need-supportive teaching approaches into student performance-related learning (33) underlined the importance of teacher feedback and peer cooperation to promote intrinsic motivation. These findings point to schools as key contexts where structured PA can be a part of that dual effect on mental health, social belonging, and educational outcomes, as long as learning environments remain supportive rather than performance-driven.

#### 3.4.3 Dance and arts-based supervised programs

However, while much of the research has focused on small, often qualitative interventions, there are indications that creative, movement-based programs can also play a role in adolescents, particularly girls struggling with internalizing difficulties, in regulating their emotions. Participants in group dance sessions articulated feelings of freedom, emotional release, and social acceptance (36) as indicated in a qualitative study. The programs, while structured rather than competitive, emphasized expression and social support and were associated with feelings of psychological safety and empowerment. Collectively, these findings suggest that supervised arts and dance-based activities can supplement traditional sport by offering low-pressure, emotionally restorative contexts.

#### 3.4.4 Domain-specific habitual activity and unstructured leisure

Researchers who study habitual PA over domains or in self-directed leisure contexts show associations associated with mental health outcomes that are at once positive and heterogeneous. Leisure-time activity, in comparison to transport or school, appeared related to affective wellbeing (37), especially in an autonomous motivation context (37). Murphy et al. (32) recognized qualitative themes of meaningful experience and self-expression emerging from mixed contexts that combined physical challenge with social interaction. This is to say that unstructured PA may nurture autonomy, identity formation, and intrinsic motivation, although the intensity and regularity of these activities differ immensely. A measure of greater motivational regulation—rather than external structure—is at the core of maintaining mental health benefits in these settings.

#### 3.4.5 Nature-based and outdoor public recreation spaces

Research that associated PA with a natural or open area consistently reported restorative and social contributions. High-quality green and public recreation spaces enhanced adolescents' connection, relaxation, and emotional recovery (34, 35). These associations, however, tended to be mediated by perceived autonomy and social integration, implying a mechanism mediated via stress restoration and attention, as opposed to direct fitness effects. Though predominantly observational, the aggregate of quantitative and qualitative results provides additional support for the conclusion that environmental context is an active driver of psychological wellbeing.

#### 3.4.6 Cross-cutting moderators and integrated trends

In organized as well as unorganized conditions, several moderators were found, having general effects on outcomes. Differences by sex were present, and girls had significantly stronger affective responses to supportive, low-stress settings, e.g., dancing or nature-based contexts, whereas boys benefited more from competitive team environments. Socioeconomic standing influenced access to organized sport, as did Badura et al. (24); Badura et al. (26), in which costs and parental availability were thought to be barriers to organized leisure activities. The benefits of unstructured outdoor play, meanwhile, were neighborhood- and environmental-specific. Lastly, motivational climate and inclusiveness proved to be universal determinants: the programs that promoted autonomy, competence, and social belonging—regardless of structure—provided the most reliable positive mental-health outcomes.

#### 3.4.7 Summary patterns

More generally, structured PA contexts offered clearer, competence-based pathways to enhanced wellbeing, whereas unstructured contexts promoted autonomy and stress recovery. The two modes are additive, not conflicting, lending greater support for a portfolio perspective in schools and communities. Synthesis of evidence suggests positive directionality across about 80% of the associations across outcomes, with negligible evidence of harm outside elite competitive contexts. These findings reinforce the idea that context is an active ingredient shaping the mental-health benefits of adolescent physical activity.

### 3.5 Additional analyses (subgroup and sensitivity patterns)

To assess heterogeneity by context, subgroup, and sensitivity analyses were performed, concentrating on population characteristics, equity-related variables, and study design robustness. As such, these analyses were intended to explain for whom and under what circumstances various PA contexts best facilitate adolescent mental health.

#### 3.5.1 Subgroup patterns

Comparing subgroups, there were some consistent demographic and situational trends found to be present. Sex differences were some of the most commonly reported moderators. Both boys and girls showed psychological benefits related to sport participation in large-scale surveys [e.g., (21, 39)]. Still, girls presented greater gains in wellbeing and self-esteem in comparison to boys in the extent to which activities were competence-based vs. performance-focused, in particular. On the other hand, boys in competitions were found to report more enjoyment of and engagement in sporting or team sports. Sexual minority youth reported different experiences within sport facilities. Team-sport participation was reported to be associated with fewer depressive symptoms, but with weaker or inconsistent effects on suicidal ideation, depending on the inclusiveness of the climate (28). This highlights the importance of clearly defining supportive settings to achieve equitable access to mental health benefits.

Age-band differences (10–14 vs. 15–19 years) indicated that younger adolescents derived more benefit-value from unstructured, autonomy-rich activities, such as play and outdoor recreation, whereas adolescents in the older age group showed more meaningful effects and positive self-concept as a result of organized sport or supervised programs (10, 11). Pathways to elite and weight-sensitive sport were a repeated risk subset. Evidence, such as Lundqvist et al. (31), identified the higher burden of stress, body image concerns, and anxiety in lean, performance-focused sport high school athletes, stressing the necessity for psychosocial support. However, excluding these elite subgroups from structured-sport analyses somewhat served to strengthen the relatively larger, positive association between sport participation and wellbeing, and these samples might counteract population-level benefits.

Finally, socioeconomic and neighborhood factors moderated access and consequences. According to Badura et al. (24) and Badura et al. (26), inequalities exist in organized leisure participation, with low-SES youth being less engaged in structured PA. Unstructured and community-based opportunities, especially in safe public recreation spaces (34, 35), partially mitigated these differences through the provision of accessible opportunities for social connectivity and stress relief. These subgroup findings should be interpreted as exploratory and hypothesis-generating, given the limited number of contributing studies and between-study heterogeneity.

#### 3.5.2 Sensitivity analyses

Analyses: The narrative synthesis was subjected to several sensitivity checks to test the robustness of the narrative synthesis. After excluding studies with elite or weight-sensitive sport samples, the proportion of positive associations was only marginally higher in structured contexts, and this pattern was consistent with the possibility that competitive pressure may attenuate observed mental health associations. Emphasizing studies that controlled for key confounders (sex, age, SES) also produced more consistent results, specifically regarding depressive and anxiety outcomes. For a given construct of both cross-sectional and longitudinal data, longitudinal evidence was preferred, as is the case for Guo and Liang (38) and Timonen et al. (40). Such analyses proved that regular PA participation prospectively predicted resilience and lower risk for later mental health disorders, providing temporal evidence for cross-sectional trends. Finally, comparing studies with respect to good design and how their motivation aspects were measured, it was observed that studies reporting a strong influence of psychological mediators (e.g., self-efficacy, autonomy, relatedness) reported stronger and more interpretable associations. This further supports the conceptual model that contextual as well as motivational elements mediate the linkage of PA with mental health, not activity dose alone.

### 3.6 Summary of certainty of evidence

At the outcome-within-context level, certainty of evidence was evaluated through the GRADE and GRADE-CERQual frameworks to ensure consistency of analysis of both quantitative and qualitative evidence. For quantitative evidence, which formed many included studies, certainty was at a range from very low to moderate. Ratings were primarily downgraded based on cross-sectional designs, self-reported exposures and outcomes, and limited control of confounding variables. The directionality was consistent across multiple independent studies [e.g., (21, 24, 26, 27, 39)] supporting moderate confidence in positive associations between structured physical activity and higher levels of wellbeing, self-concept, and resilience. Longitudinal studies and quasi-experimental data [e.g., (38, 40)] provided stronger evidence consistent with potential causal associations, thereby supporting higher certainty ratings for resilience-related and mental health outcomes. Yet general confidence was still limited by variations in the measures and incomplete correction for socioeconomic and motivational factors. In terms of qualitative evidence, the CERQual appraisal reflected moderate confidence in themes derived from unstructured, nature-based, and arts-related environments [e.g., (32, 34, 36)]. Emotional restoration, autonomy, and social connection were common themes through unstructured or outdoor participation across the studies.

Overall, methodological rigor, coherence of themes in diverse contexts, and adequacy of sample representation were high; the potential transferability of results was limited, however, by geographic concentration and small numbers of participants. This integration of types of evidence indicates that, though the possible magnitude of effects is not fully estimated, both direction and consistency of results suggest that physical-activity context may exert meaningful effects on adolescent mental health. Structured activities were consistently associated with higher levels of competence and relatedness, whereas unstructured and nature-based contexts were more frequently associated with autonomy and psychological restoration. Consistent with this, the certainty of evidence for structured PA contexts was rated moderate in wellbeing and self-concept, low to moderate in resilience and depressive symptoms, and very low for suicidal ideation outcomes, because longitudinal data were sparse. For unstructured and nature-based contexts, certainty was moderate for perceived restoration and social connectedness, but low for quantitative indicators owing to limited means of standardized measurement. These analyses have collectively provided a rationale for framing the contextualization of mental-health benefits in adolescence, while future research should enhance causality by implementing multi-wave/experimental designs, aligning outcome metrics, and reporting motivational and environmental factors in an unambiguous manner.

Overall certainty ranged from very low to moderate, given the predominance of observational designs and reliance on self-report measures. Across studies, findings predominantly reflect observed associations rather than causal effects. Contextual moderators, including supervision, environmental quality, and autonomy support, were frequently underreported (see Supplementary Table S5).

## 4 Discussion

A portfolio approach that combines supervised competence-building with accessible, autonomy-rich opportunities aligns with public-health delivery in schools and municipalities. Policies should mandate inclusive team climates and low-cost access and specify environmental quality and safety indicators for public spaces.

### 4.1 Interpretation of main findings across contexts

This review integrates data from 22 studies with varying areas of adolescent PA. Taken together, the findings do support the idea that context indeed counts: formal involvement in organized sport, school programs, and supervised arts activities is generally correlated with greater wellbeing, resilience, and self-concept while unstructured or natural contexts present distinct attributes of autonomy, restoration, and social connectedness (17, 19, 24, 26, 39, 44, 45). However, the available evidence remains insufficient to draw firm conclusions regarding the presence or absence of potential adverse mental health effects outside elite or weight-sensitive sport contexts, as most studies did not explicitly assess harm-related outcomes. The general tendency is in accordance with the contextual-mechanistic theory in which structured practice is primarily concerned with the competence and relatedness domains, and unstructured practice is concerned with autonomy and environmental restoration (10–13, 21–26). At the same time, while the present synthesis organizes evidence by treating physical activity context as the primary analytic dimension, it is important to recognize that the relationship between physical activity and mental health is likely bidirectional, as adolescents' psychological status may also influence participation patterns, particularly in observational studies.

Although most included evidence remains cross-sectional and therefore cannot establish temporal ordering, multi-wave longitudinal studies using cross-lagged panel approaches and objective indicators (for example, device-based step counts) can better evaluate reciprocal dynamics while reducing self-report bias. However, many such studies operationalize physical activity as overall movement rather than distinguishing structured and unstructured contexts. As a result, despite their methodological strengths for directionality, they could not be integrated into the present context-classified synthesis. Future context-focused research would benefit from pairing objective PA measurement with explicit contextual classification to strengthen both causal interpretation and setting-specific translation.

From an evidential hierarchy perspective, the synthesis is predominantly informed by cross-sectional studies, which consistently support associative relationships but do not establish temporal ordering. The small number of longitudinal or quasi-experimental studies offers more informative temporal insight, yet remains insufficient to draw firm conclusions regarding directionality across physical activity contexts. Accordingly, interpretations in this review are framed in terms of observed associations rather than causal effects. This distinction is particularly relevant for outcomes such as depression, anxiety, and psychosocial functioning, where longitudinal evidence remains limited. Future research would benefit from multi-wave designs that explicitly classify activity context while integrating objective physical activity measurement.

### 4.2 Mechanistic and theoretical considerations

Our synthesis supports the results of the prediction of Self-Determination Theory (SDT) (12, 14, 21). Across team sport, school physical education, and dance interventions, adolescents with higher levels of need satisfaction, especially autonomy support and perceived competence, indicated higher life satisfaction and psychological vitality (11, 21, 46). In contrast, controlling or evaluative motivational climates, as seen in elite or weight-sensitive sport programs, were identified with stress and poor wellbeing (35). Nevertheless, evidence regarding whether such motivational climates are associated with adverse outcomes outside elite contexts remains limited. The current results are consistent with motivational-regulation models highlighting the importance of satisfying psychological needs as a determinant of long-term engagement and the benefits for mental health, rather than increasing activity dosage (14, 15, 39). The complementary theories of stress recovery and attention restoration can help explain the observed improvements in emotional regulation and felt connectedness associated with unstructured outdoor and nature-based activities (17, 19, 44). Qualitative information on relaxation, freedom, and a feeling of community in natural spaces (44) is analogous to experimental data showing that environmental exposure is associated with lower levels of physiological stress and cognitive fatigue (8, 9, 13). As a result, the mental-health benefits of PA cannot be separated from their physical or social context.

### 4.3 Cross-context synthesis and complementarity

Informed by both structured and unstructured paradigms, this review adds to prior dose-based reviews (7, 9, 15) and shows context to be an active ingredient of PA's psychological effects. Autonomy-supportive environments support competence development, goal mastery, and peer belonging (21, 24, 47), whereas unstructured contexts support flexibility, identity exploration, and self-expression (39, 42, 48). Importantly, these associations should be interpreted within a likely bidirectional framework, whereby adolescents' mental health may both influence and be influenced by participation in different physical activity contexts (49). Taken together, these patterns suggest potential complementarity between contexts; however, the current evidence base provides limited information on potential harms and remains insufficient to conclude whether specific contexts are protective or harmful for particular adolescent subgroups. Such complementarity suggests that public-health and educational strategies should combine supervised structure with accessible, autonomy-rich opportunities, consistent with ecological models that frame adolescent health as co-determined by personal agency and environmental affordances (8, 16, 24).

### 4.4 Equity, access, and policy implications

While on the whole it is better, there remain some imbalances. Low-SES children are underserved in organized sport (26, 30), and marginalized or sexual minority youth, in teams, can encounter exclusion or stigmatization (50). On the other hand, unstructured or community-based options, including parks, public recreation spaces, and outdoor leisure, are found to be more equitable. Still, the degree of equity is in turn tied to neighborhood safety and environmental quality (44, 45). Although structured programs may offer psychosocial benefits, the evidence regarding unintended or adverse effects remains limited, underscoring the need for cautious interpretation and ongoing monitoring in policy implementation. Therefore, context-inclusive policy should prioritize a small number of actionable recommendations, including ensuring equitable access to quality, autonomy-supportive organized sport and structured programs, improving the availability and safety of public spaces that facilitate free play and nature-based activity, and integrating motivational principles, such as autonomy support and inclusiveness, into school physical education curricula. In addition, systematic monitoring of socioeconomic and contextual inequalities in access to physical activity opportunities is warranted. These policy and practice implications are largely informed by observational evidence and should be interpreted with caution, but they are consistent with international adolescent health frameworks that emphasize integrated, context-sensitive approaches to mental health promotion (1–6).

### 4.5 Strengths and limitations

(1) Methodological strengths

From a methodological perspective, this review demonstrates several strengths. The systematic search and study selection followed PRISMA guidelines, with dual independent screening and data extraction to minimize selection bias. The use of multiple, design-appropriate quality appraisal tools (JBI, ROBINS-I, and CASP-Qualitative) allowed for a nuanced assessment of risk of bias across heterogeneous study designs. In addition, the combined application of GRADE and CERQual enhanced transparency in evaluating the certainty of quantitative and qualitative evidence, supporting balanced interpretation of findings (17, 19, 44).

(2) Conceptual and applied strengths

Conceptually and in terms of applied relevance, this review is strengthened by its explicit focus on physical activity context as a primary analytic dimension. By differentiating structured and unstructured forms of participation, the synthesis moves beyond dose-based paradigms and integrates mechanistic, psychosocial, and environmental perspectives into a coherent framework (8, 14–16). The inclusion of equity-related considerations, such as socioeconomic status, gender, and sexual minority status, further enhances the applied relevance of the findings. In addition, the emphasis on policy and practice implications aligns with international public health priorities that promote integrated physical and mental health strategies for adolescents (1–6).

(3) Limitations

Several limitations should be considered when interpreting these findings. First, the predominance of cross-sectional and observational study designs limits causal inference, as only a small number of studies employed longitudinal or quasi-experimental approaches (30, 51). Accordingly, directional inference is limited for the majority of included studies, as most evidence derives from cross-sectional designs, with only a small number of longitudinal or quasi-experimental studies allowing more tentative inferences regarding temporal ordering. Second, heterogeneous operational definitions of structured and unstructured contexts were used across studies; in some cases, distinctions between domain-specific physical activity (e.g., leisure vs. transport) and motivational or organizational characteristics were blurred. Third, most studies relied on self-reported measures of physical activity exposure and mental health outcomes, which may introduce reporting bias and limit measurement accuracy. The limited availability of device-based measurement within context-classified studies further constrained inferences about directionality and measurement precision. Fourth, gray literature was not included, which may have contributed to publication bias by preferentially capturing studies with statistically significant or positive findings. Fifth, the evidence base is dominated by studies conducted in high-income Western countries, with limited representation from low- and middle-income settings, potentially restricting generalizability (24, 26, 39, 44). Selection bias cannot be ruled out, as adolescents who are more physically active or motivated may have been more likely to participate in the included studies. Moreover, the inconsistent reporting of adverse mental health outcomes further limits the ability to conclude whether specific participation contexts are protective or potentially harmful for certain adolescent populations. Finally, the review was not prospectively registered, which may limit transparency regarding *post hoc* methodological refinements and increase the risk of perceived selective reporting, despite the use of established methodological frameworks.

### 4.6 Future directions

First, subsequent research should seek to disentangle the causal route to physical activity context-related mental health from robust longitudinal or intervention studies. Research that utilizes multilevel modeling and repeated measures might have superior temporal relationships and differentiate temporal short-term affective responses from long-term psychological adaptation (30, 48, 51). Combining objective measures with psychophysiological and ecological variables (e.g., HRV, perceived autonomy, environmental exposure) would likely strengthen construct validity (7, 9, 45). Second, future work should focus on hybrid mechanistic-implementation theories that integrate behavioral science and ecological psychology (14–16). These models can indicate mediators (autonomy, competence, relatedness, restoration) and moderators (SES, gender, environmental quality) that drive intervention response. Mixed-methods designs are essential to gather context, while the inclusion of young people's perspectives, as well as the contextualization, is quite helpful. Third, a wide range of studies focused on equity, in response to the needs and traditions of communities, is sorely needed. Studies in underrepresented areas should investigate the way public space design, school infrastructure, and community norms shape participation and wellbeing outcomes (24, 44, 52).

Comparative analysis in different socioeconomic groups can be used to determine how policy should address access to well-structured programming in conjunction with investments in inclusive community recreation (26, 30, 53). It may be valuable to experiment with context-integrated policy models that integrate structured school initiatives with community-based or digital leisure platforms, as such models may help support more sustainable investment across a wide range of youth populations (3, 5, 8, 15). Incorporating participatory modalities, where adolescents co-design interventions, may further enhance ownership, relevance, and long-term adherence (12, 14, 39).

## 5 Conclusion

In conclusion, this systematic review highlights physical activity context as a central factor shaping adolescents' mental health experiences, with structured and unstructured settings offering distinct and potentially complementary psychosocial affordances. Interpretation of these findings is constrained by the predominance of observational designs, reliance on self-reported measures, and inconsistent specification of contextual and motivational characteristics. Future research should prioritize longitudinal and experimental studies, clearer operationalization of activity context and motivational climate, and the use of more standardized and objective mental health indicators, to strengthen causal inference and support context-sensitive intervention design. Within these limitations, the findings underscore the relevance of considering physical activity context when informing public health strategies aimed at promoting adolescent mental health.

## Data Availability

The original contributions presented in the study are included in the article/Supplementary material, further inquiries can be directed to the corresponding authors.
