# Supplementary material for: Physical activity contexts and adolescent mental health: a systematic review of structured and unstructured approaches, 2015–2025
Source: Front Public Health. 2026 Mar 30;14:1737783. doi: 10.3389/fpubh.2026.1737783 (PMC13070774; doi:10.3389/fpubh.2026.1737783)
Supplement: Supplementary file 1 [file Table_1.docx]

**Supplement S1 PRISMA 2020 Checklist (Tailored to Physical activity contexts and adolescent mental health)**

| **Section** | **Item** | **Checklist description** | **Location in manuscript** |
| --- | --- | --- | --- |
| **TITLE** | 1 | Identify the report as a systematic review. | Title page: “... a systematic review of structured and unstructured approaches” |
| **ABSTRACT** | 2 | Structured summary of objectives, methods, results, and conclusions. | Abstract, p. 1 |
| **INTRODUCTION** | 3 | Describe the rationale for the review in the context of existing knowledge. | 1.1 Rationale, p. 2 |
|  | 4 | Provide an explicit statement of the objectives. | End of Introduction, “Objective” paragraph, p. 3 |
| **METHODS** | 5 | Specify inclusion and exclusion criteria using the PECOD framework. | 2.1 Eligibility Criteria, p. 4-5 |
|  | 6 | Specify information sources (databases, date of last search). | 2.2 Information sources, p. 5 |
|  | 7 | Present the full search strategy (keywords, time range). | 2.2 Information sources, p. 5 |
|  | 8 | Specify methods used to select studies (screening process). | 2.3 Study Selection, p. 5 |
|  | 9 | Describe the data collection process and the variables extracted. | 2.4 Data Extraction, p. 5-6 |
|  | 10 | List and define all outcomes and variables sought. | 2.4 Data Extraction, p. 5-6 |
|  | 11 | Describe methods for assessing risk of bias. | 2.5 Risk of Bias Assessment, p. 6-7 |
|  | 12 | Specify effect measures or summary indicators. | 2.6 Synthesis methods, p. 7 |
|  | 13 | Describe synthesis methods (qualitative/narrative). | 2.6 Synthesis methods, p. 7 |
|  | 14 | State how heterogeneity was handled and sensitivity analyses. | 2.7 Subgroup and Sensitivity Considerations, p. 7 |
|  | 15 | Describe methods to assess certainty/confidence in evidence. | 2.8 Certainty of Evidence, p. 7 |
|  | 16 | Note registration and protocol status. | 2.9 Deviations from Protocol, p. 7 (“Not registered”) |
| **RESULTS** | 17 | Describe the number of records identified, screened, and included, with reasons for exclusions. | 3.1 Study Selection, p. 7-8; Table 2; Supplemen S6 |
|  | 18 | Provide characteristics of included studies. | 3.2 Study Characteristics, p. 8-9; Supplement S4 |
|  | 19 | Present risk of bias assessments for each study. | 3.3 Risk of Bias, p. 9-10; Table 1; Figure 2 |
|  | 20 | Summarize results for each outcome/domain. | 3.4 Synthesis by Context and Outcome, p. 10-12; Figure 3; Table 3 |
|  | 21 | Present subgroup or sensitivity analyses. | 3.5 Additional Analyses, p. 13 |
|  | 22 | Present certainty of evidence. | 3.6 Summary of Certainty of Evidence, p. 14-15 |
| **DISCUSSION** | 23 | Summarize the main findings and the strength of evidence. | 4.1–4.5, p. 15-16 |
|  | 24 | Discuss limitations of evidence and of the review process. | 4.5 Strengths and Limitations, p. 16-17 |
|  | 25 | Provide general interpretation and implications. | 4.6, and 5. Conclusions, p. 17-18 |
| **OTHER INFORMATION** | 26 | Describe funding sources and potential conflicts of interest. | Acknowledgments / Funding statement section, p. 18 |
|  | 27 | Provide a data availability statement. | “Data sharing not applicable…” (p. 23) |
