# Supplementary material for: Physical activity contexts and adolescent mental health: a systematic review of structured and unstructured approaches, 2015–2025
Source: Front Public Health. 2026 Mar 30;14:1737783. doi: 10.3389/fpubh.2026.1737783 (PMC13070774; doi:10.3389/fpubh.2026.1737783)
Supplement: Supplementary file 2 [file Table_2.docx]

**Supplement S2. Information sources and search strategy summary**

| **Database** | **Platform / Provider** | **Coverage period** | **Search fields** | **Sample search string (see Supplement S1 for full)** |
| --- | --- | --- | --- | --- |
| **PubMed** | U.S. National Library of Medicine (NIH) | January 2015–October 2025 | Title, Abstract, MeSH Terms | (“physical activity” OR exercise OR sport OR “leisure time activity”) AND (adolescent OR youth OR teen) AND (“mental health” OR depression OR anxiety OR well-being OR resilience) |
| **Web of Science Core Collection** | Clarivate Analytics | January 2015–October 2025 | Topic (Title, Abstract, Keywords) | (“physical activity” OR exercise OR sport) AND (adolescent OR youth) AND (“mental health” OR well-being OR resilience) |
| **Scopus** | Elsevier | January 2015–October 2025 | Title, Abstract, Keywords | (“physical activity” OR exercise OR sport) AND (adolescent OR youth) AND (“mental health” OR depression OR anxiety OR well-being) |
| **PsycINFO** | EBSCOhost / American Psychological Association | January 2015–October 2025 | Title, Abstract, Subject Headings | (“physical activity” OR exercise OR sport) AND (adolescent OR youth) AND (“mental health” OR affective OR emotional OR psychosocial) |

**Note.** Search strings were adapted for each database using controlled vocabulary (e.g., MeSH, Thesaurus terms) and Boolean operators. Reference lists of included studies and relevant reviews were manually screened to identify additional records. The complete search syntax and iteration details are presented in *Supplementary Table S1*.
