# Supplementary material for: Physical activity contexts and adolescent mental health: a systematic review of structured and unstructured approaches, 2015–2025
Source: Front Public Health. 2026 Mar 30;14:1737783. doi: 10.3389/fpubh.2026.1737783 (PMC13070774; doi:10.3389/fpubh.2026.1737783)
Supplement: Supplementary file 3 [file Table_3.docx]

**Supplement S3. Data extraction framework**

| **Category** | **Variables extracted** | **Operational notes** |
| --- | --- | --- |
| Bibliographic information | Author(s), year, journal, country | Used for regional and temporal mapping of evidence |
| Population characteristics | Sample size (N), mean age or range (10–19), sex distribution, population subgroup (e.g., elite athletes, sexual minority youth) | Classified as general, school, clinical, or special subgroup samples |
| Physical activity (PA) context | Structured (organized sport, team sport, PE, classroom activity breaks, supervised arts/dance) or unstructured (leisure, outdoor, public recreation, self-directed) | Context classification followed the operational definitions established in the review protocol |
| Setting | School, community, clinical, or elite/high-performance environment | Recorded to identify ecological or implementation variability |
| Exposure operationalization | Participation frequency, duration, type, motivation climate, or environmental exposure | Extracted as defined in each study, with standardization notes where applicable |
| Comparator | Non-participants, alternative PA context, or within-sample domain contrast | Recorded verbatim from the study design and adjusted for the direction of association |
| Outcomes and measures | Mental-health and psychosocial constructs: depression, anxiety, well-being, self-concept, resilience, prosocial behavior, connectedness | Measurement tools (e.g., PHQ-9, SDQ, WHO-5, RSES, RS-14, bespoke scales) were documented |
| Analytic approach | Statistical or qualitative methods (e.g., regression, mediation, cross-lagged model, thematic analysis) | Noted for the synthesis of methodological diversity |
| Effect direction and size | Direction (positive, null, negative) and effect estimates (β, OR, r, or qualitative strength) where available | Quantified or coded for inclusion in the effect-direction plot |
| Moderators and covariates | Sex, age, socioeconomic status, motivation climate, and environmental factors | Used in subgroup and sensitivity analyses |
| Implementation or contextual notes | Inclusion climate, evaluation pressure, supervision quality, and resource accessibility | Summarized for equity and feasibility interpretation |
| Funding and conflicts | Reported funding source and conflict declarations | Recorded for transparency and potential bias assessment |
| Risk of bias indicators | Appraisal tool used (JBI, ROBINS-I, CASP), overall RoB judgment | Corresponds to Table 2 and Figure 3 |

Note. Data were extracted independently by two reviewers using a piloted codebook (Supplement S3). Discrepancies were reconciled through discussion and consensus.
