# Supplementary material for: Physical activity contexts and adolescent mental health: a systematic review of structured and unstructured approaches, 2015–2025
Source: Front Public Health. 2026 Mar 30;14:1737783. doi: 10.3389/fpubh.2026.1737783 (PMC13070774; doi:10.3389/fpubh.2026.1737783)
Supplement: Supplementary file 4 [file Table_4.docx]

**Supplementary S4 Study characteristics**

| **Ref. No.** | **Author (Year)** | **Sample Size** | **Country/Region** | **Study Type** | **Setting** | **Age Range** | **PA Type** | **Key Mental Health Outcomes** | **Main Findings** |
| --- | --- | --- | --- | --- | --- | --- | --- | --- | --- |

| [10] | Fraguela-Vale et al. (2020) | 129 secondary-school students | Spain | Cross-sectional motivational study | School PE and extracurricular PA | ≈14–16 yrs | PE, organized sport, leisure-time PA | Basic psychological needs, physical self-concept | Autonomy satisfaction in PE and active leisure was the strongest predictor of physical self-concept and PA, showing that autonomy-supportive PA environments enhance adolescents’ psychological functioning. |
| --- | --- | --- | --- | --- | --- | --- | --- | --- | --- |
| [11] | Valero-Valenzuela et al. (2021) | 618 PE students | Spain | Cross-sectional SDT-based study | Secondary-school PE | 10–14 yrs | PE tasks and extracurricular PA | Physical self-concept, autonomous motivation, vitality | Autonomous motivation and need satisfaction in PE predicted better physical self-concept, supporting SDT-consistent PE teaching and active tasks. |
| [21] | Guddal et al. (2019) | 7,619 adolescents (Young-HUNT) | Norway | Population-based cross-sectional survey | School and community | 13–19 yrs | General PA, team sport, organized sport | Psychological distress, self-esteem, and life satisfaction | Higher PA and team-sport participation were associated with higher self-esteem and life satisfaction and lower psychological distress, especially among older girls. |
| [24] | Baďura et al. (2021) | 55,429 adolescents (HBSC 2017/2018) | Europe (8 countries)+ Canada | Population survey on organized leisure-time activities | School and community, nationally representative | 11–15 yrs | Organized leisure-time PA/sport | Life satisfaction, multiple health complaints | Participation in organized leisure, mainly sport, was associated with higher life satisfaction and fewer complaints across SES groups, although access inequalities remained. |
| [26] | Badura, Svacina & Hallingberg (2024) | 14,128 (European adolescent survey) | Europe (Czech) | Cross-sectional study on enjoyment vs obligation in OLTA | School and community leisure-time settings | 11–15 yrs | Organized leisure-time PA and sport | Subjective health, life satisfaction, psychosomatic complaints | Adolescents who enjoyed organized PA and sport reported better mental health and well-being, even when they also felt some obligation to attend, so programs should prioritize enjoyment. |
| [27] | Chi (2022) | 1,714 (approx.; CFPS adolescents) | China | Cross-sectional (national survey) | Community/household survey | Adolescents (12–17 years) | Structured (sports participation) | Depressive symptoms; anxiety disorder | Sports participation is associated with lower odds of depression and anxiety among adolescents. |
| [28] | Larocca et al. (2022) | 46,537 LGBTQA adolescents | United States | Cross-sectional secondary analysis | School setting, sexual and gender minority youth | 14–18 yrs | Team-sport participation (school or community) | Depressive symptoms, suicidal ideation | Team-sport participation was linked to lower depression among LGBTQA youth, while associations with suicidal ideation were weaker and subgroup dependent, indicating the need for inclusive team climates. |
| [29] | Latino et al. (2023) | 100  middle-school students | Italy | School-based quasi-experimental study | Classroom-based PA embedded in lessons | 14–15 yrs | Structured classroom PA breaks | Self-efficacy, academic achievement | Short, structured classroom PA improved self-efficacy and school performance in both normal-weight and overweight youth. |
| [30] | Ortega-Gómez et al. (2023) | 268 adolescents | Spain | Cross-sectional school-based study | Secondary schools, DADOS study | aged 13.9 ± 0.3 years | Habitual PA and health-related fitness | Self-confidence, interpersonal relations | Higher PA and fitness were associated with higher self-confidence and better interpersonal relations. |
| [31] | Lundqvist et al. (2023) | 8 (elite adolescent athletes) | Sweden | Qualitative interviews within the intervention context | Sports high schools; elite lean athletes | Late adolescents (16-18 years) | Structured (elite sport) | Mental health concerns; need for psychosocial support | Psychosocial athletes at sports high schools face multiple mental health risks; psychosocial support is required. |
| [32] | Murphy et al. (2022) | 58 (13 focus groups) | Ireland | Qualitative focus groups | Schools/community contexts | Adolescents (16-18 years) | Mixed (contexts of PA & sport) | Perceived mental health & wellbeing; self-esteem | Meaningful, choice-rich, and socially supportive PA contexts are perceived to enhance mental health. |
| [33] | Cronin et al. (2022) | 309  youth-sport participants | United Kingdom | Cross-sectional, SDT-based | Community and school youth-sport programs | 11–18 yrs | Structured, coach-led youth sport | Need satisfaction, life-skills development, well-being | Autonomy-supportive coaching promoted life skills and well-being, whereas controlling coaching undermined these benefits. |
| [34] | Puhakka & Hakoköngäs (2023) | 39 adolescents | Finland | Qualitative study (photo-elicitation) | Every day contact with nearby nature | 15–16 yrs | Unstructured nature/outdoor experiences | Everyday well-being, relaxation, social connectedness | Nature was described as an easy, frequent source of everyday well-being through calmness and socializing outdoors. |
| [35] | Rivera et al. (2022) | visitation (n = 349); physical activity (n = 441) adolescents | Australia | Cross-sectional survey | Use of outdoor public recreation spaces | aged 15.4 ± 1.6 years yrs | Outdoor public recreation spaces and activity | Social connectedness | Adolescents who reported good access to and use of outdoor recreation spaces also reported stronger social connectedness. |
| [36] | Duberg et al. (2016) | 112 (girls) | Sweden | Qualitative study embedded in RCT | School/community dance intervention | Adolescent girls (13-18 years) | Structured (dance sessions) | Internalizing symptoms: emotional well-being | Participants reported feelings of freedom, social support, and improved well-being through dance. |
| [37] | White et al. (2018) | 1,632 | Australia (Western Sydney) | Observational; structural equation modeling | Government-funded high schools (14 schools) | Mean 12.94 years (≈12–14) | Mixed (leisure-time & active travel) | Affective well-being (positive/negative affect) | Leisure-time PA linked to higher positive and lower negative affect; active travel effects depended on autonomous motivation. |
| [38] | Guo & Liang (2023) | 818 | China | Two-wave cross-lagged panel | Secondary schools (grades 7–8 and 10–11) | 12–17 years | Mixed (habitual PA; domain-unspecified) | Resilience | PA exerted prospective effects on resilience; relational asymmetry favored PA resilience. |
| [39] | Liu et al. (2023) | 67,281 school-attending students | China | Large cross-sectional school survey | Primary, junior, and senior secondary schools | Adolescent  ≈10–18 yrs | Organized sport participation (frequency, type) | Subjective well-being, life satisfaction | Any sport participation was associated with higher subjective well-being, and frequent or team-based participation showed the strongest associations, independent of sex and weight. |
| [40] | Timonen et al. (2021) | 6,838 adolescents with follow-up | Finland | Longitudinal cohort study | School-based survey with registry linkage | 15–16 yrs at baseline, followed to early 20s | Social leisure and organized activities | Onset of mental disorders in young adulthood | Adolescent participation in social leisure activities predicted a lower risk of diagnosed mental disorders in young adulthood. |
| [41] | Hui et al. (2022) | 1,053  junior-middle-school students | China | Cross-sectional survey with chain mediation | Junior middle schools, post-epidemic period | 12–15 yrs | Regular physical exercise (frequency and intensity) | Prosocial behavior, emotional intelligence, sports learning motivation | Physical exercise improved prosocial behavior indirectly through higher emotional intelligence and stronger sports learning motivation, with gender differences. |
| [42] | Wheatley et al. (2020) | 7,385  British state secondary schools | United Kingdom | Cross-sectional baseline analysis (cluster-RCT cohort) | Secondary schools, Fit to Study trial | 11–13 yrs | Cardiorespiratory fitness and habitual PA | Mental-health difficulties, subjective well-being | Higher fitness and higher PA were both associated with better mental health and well-being in young British adolescents. |
| [43] | Rueth & Lohaus (2022) | 1,727 (pre-)adolescents | Germany | Cross-sectional, process-oriented measurement | School-based, general adolescent context | 9–18 yrs | Not PA-specific, emotion-regulation processes | Psychosocial adjustment, well-being, and emotion-regulation success | Better emotion regulation was associated with better psychosocial adjustment, supporting ER components in school PA/well-being programs. |
