# Supplementary material for: Physical activity contexts and adolescent mental health: a systematic review of structured and unstructured approaches, 2015–2025
Source: Front Public Health. 2026 Mar 30;14:1737783. doi: 10.3389/fpubh.2026.1737783 (PMC13070774; doi:10.3389/fpubh.2026.1737783)
Supplement: Supplementary file 5 [file Table_5.docx]

**Supplement S5 Summary of certainty of evidence across outcome domains**

| **Outcome domain** | **Context(s)** | **Predominant study design(s)** | **Certainty rating** | **Key determinants** | **Interpretation** |
| --- | --- | --- | --- | --- | --- |
| **Depression and anxiety** | Structured (sport, PE, dance); Unstructured (leisure, outdoor) | Cross-sectional and longitudinal | **Moderate** | Consistent direction of association; mostly self-report measures; limited causal inference | Evidence supports a beneficial association between physical activity and lower symptoms, though temporal confidence is limited. |
| **Well-being and affect** | Structured and unstructured | Cross-sectional, intervention, qualitative | **Moderate to high** | Convergent findings across designs; supportive qualitative themes; good coherence | Strongest evidence for mood enhancement and emotional recovery, especially under autonomy-supportive conditions. |
| **Self-concept and resilience** | Structured (sport, PE) | Cross-sectional, longitudinal | **Moderate** | Consistent direction; small number of longitudinal designs; some residual confounding | Moderate confidence that structured participation enhances competence and resilience. |
| **Social connectedness and prosocial behavior** | Structured (team sport); Unstructured (community, leisure) | Cross-sectional, qualitative | **Low to moderate** | Inconsistent operationalization; small qualitative base | Suggestive but less consistent evidence for social bonding and peer integration. |
| **Stress recovery and perceived restoration** | Unstructured (outdoor, nature-based) | Qualitative | **Moderate confidence (CERQual)** | High coherence and adequacy; minor methodological limitations | High confidence that natural or self-directed contexts foster stress relief and attentional restoration. |
| **Identity and motivation** | Both contexts | Cross-sectional, qualitative | **Moderate** | Aligned with Self-Determination Theory constructs, variable measures | Consistent evidence that autonomy-supportive environments reinforce intrinsic motivation and identity formation. |

Certainty ratings are based on GRADE for quantitative evidence and GRADE-CERQual for qualitative evidence (very low, low, moderate, high). Detailed justifications are provided in Supplement S5.
