# Supplementary material for: Physical activity contexts and adolescent mental health: a systematic review of structured and unstructured approaches, 2015–2025
Source: Front Public Health. 2026 Mar 30;14:1737783. doi: 10.3389/fpubh.2026.1737783 (PMC13070774; doi:10.3389/fpubh.2026.1737783)
Supplement: Supplementary file 6 [file Table_6.docx]

**Supplement S6. Main reasons for full-text exclusion**

| **Reason category** | **Operational definition** | **n** | **%** |
| --- | --- | --- | --- |
| Wrong population or age | Did not target adolescents aged 10–19 years, or adolescent data could not be separated | 18 | 15.5 |
| No PA **context** exposure | Examined physical activity as overall volume, dose, or fitness indicators (including device-based measures) without contextual classification or comparison (structured vs unstructured, domain-specific). | 22 | 19.0 |
| No eligible mental-health outcome | Lacked depression, anxiety, well-being, self-concept, resilience, prosocial/connectedness, or allied outcomes | 20 | 17.2 |
| Not a primary empirical study | Review, commentary, conceptual article, protocol, editorial, or correspondence | 14 | 12.1 |
| Inadequate design or reporting | Insufficient detail to determine exposure–outcome relation (e.g., measures/analysis unclear) | 12 | 10.3 |
| Duplicate/overlapping dataset | Overlapped with another included report; the most comprehensive version retained | 8 | 6.9 |
| Non-English or full text unavailable | Not published in English or could not retrieve the full text | 10 | 8.6 |
| Other (specified in S2) | Miscellaneous reasons detailed in Supplement S2 | 12 | 10.3 |
| **Total** |  | **116** | **100.0** |

Note.

The category “No PA context exposure” includes studies in which physical activity was operationalized as overall activity, dose, or fitness indicators (including device-based measures), without contextual classification (e.g., structured vs unstructured). These studies were not eligible for inclusion because the exposure could not be aligned with the predefined context framework used in this review.
